# Supplementary figures and images for: March 2019 dengue fever outbreak at the Kenyan south coast involving dengue virus serotype 3, genotypes III and V
Source: PLOS Glob Public Health. 2022 Mar 24;2(3):e0000122. doi: 10.1371/journal.pgph.0000122 (PMC10021577; doi:10.1371/journal.pgph.0000122)

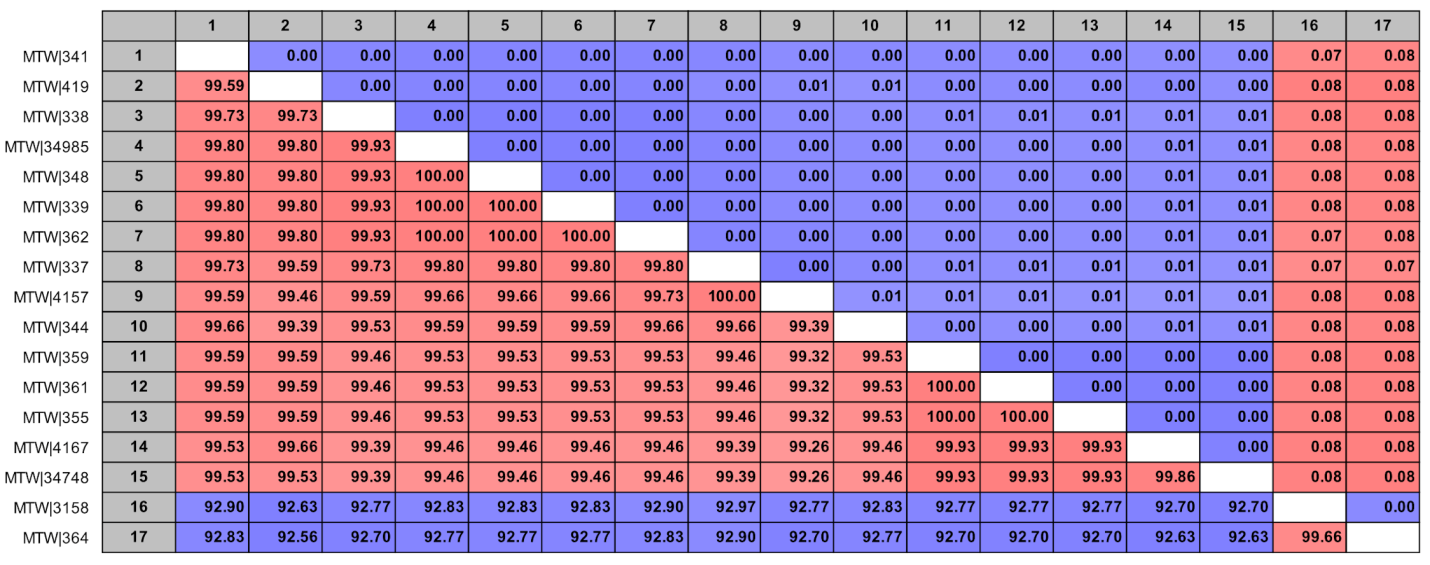

Supplement: S1 Fig — The 15 genotype III sequences (shown in orange) had a nucleotide sequence similarity > 99.3%. The other 2 genotype V samples (shown in blue) had nucleotide sequence similarity of 99.6% (S1 Fig). The genotype III and V sequences differed by 6.7%. (TIFF) [file pgph.0000122.s001.tiff]

**
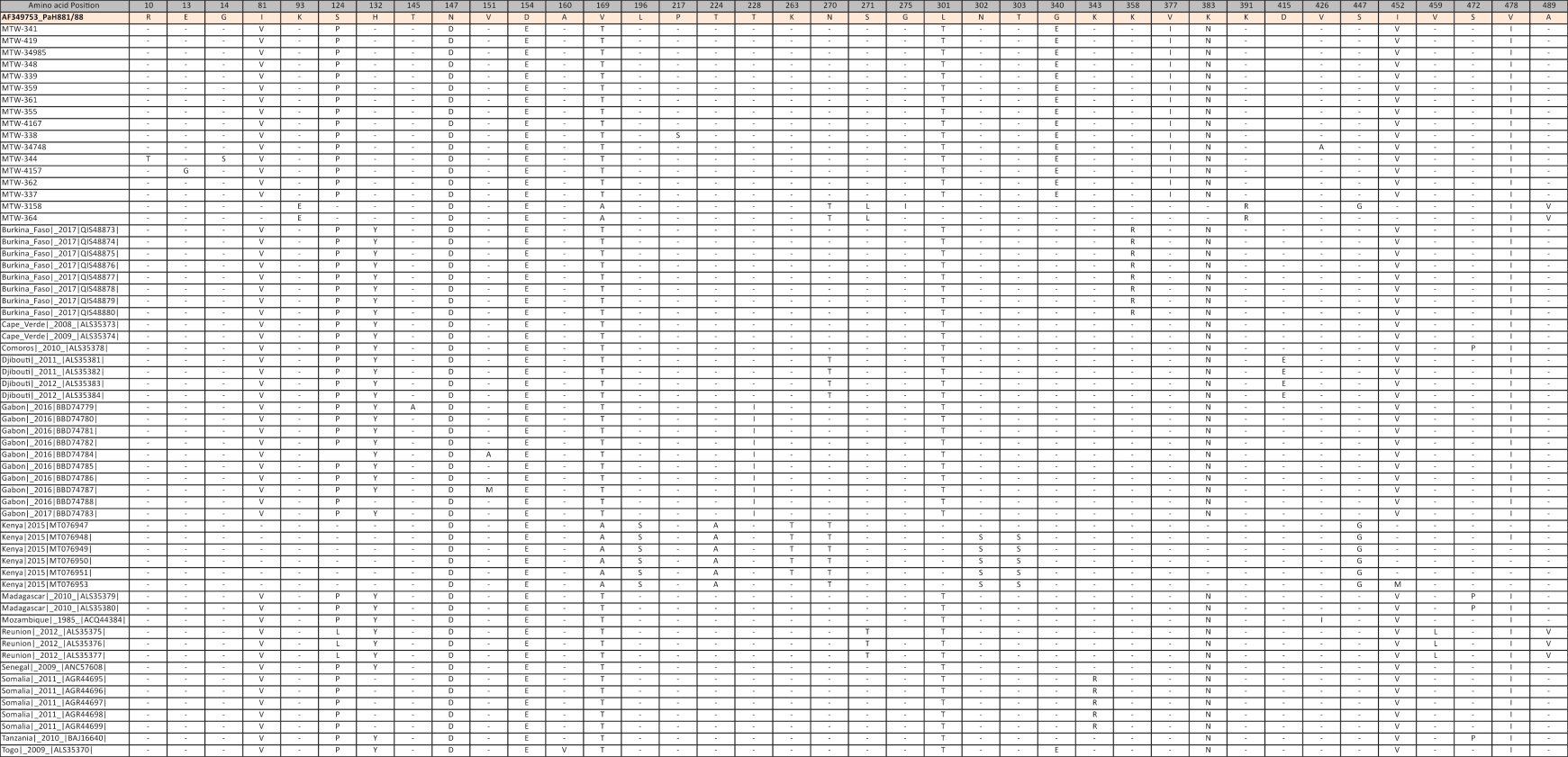
**

Supplement: S1 Table — (DOCX) [file pgph.0000122.s002.docx]
