# Supplementary material for: March 2019 dengue fever outbreak at the Kenyan south coast involving dengue virus serotype 3, genotypes III and V
Source: PLOS Glob Public Health. 2022 Mar 24;2(3):e0000122. doi: 10.1371/journal.pgph.0000122 (PMC10021577; doi:10.1371/journal.pgph.0000122)
Supplement: S2 Table — The datasets were down sampled by geographical location, year of collection, genotype and presence of the full genomes, or the presence of complete E genes. (DOCX) [file pgph.0000122.s003.docx]

Argentina|2007|EU052793|,Bolivia|2011|MH888333|,Colombia|2014|KX910787|,Colombia|2013|KY851590|,Colombia|2010|KX926490|,French_Polynesia|2013|KU053470|,Colombia|2015|MH544647|,Brazil|2004|FJ850083|,Brazil|2008|FJ850094|,Brazil|2002|KC425219|,Brazil|2003|EF643017|,Brazil|2002|JX669490|,Brazil|2003|JX669489|,Brazil|2006|GQ868548|,Brazil|2006|GU131845|,Brazil|2007|GU131878|,Brazil|2001|FJ898446|,Paraguay|2002|JF808123|,Brazil|2003|JF808124|,Brazil|2005|JX669497|,Brazil|2006|JX669507|,Anguilla|2001|FJ898462|,Barbados|2004|JN379509|,Antigua_and_Barbuda|2001|JN379489|,Barbados|1998|JF804040|,Barbados|2001|JN379488|,Barbados|2002|JN379492|,Barbados|2003|JN379497|,Barbados|2004|JN379500|,Grenada|2002|JN379506|,Grenada|2003|JN379498|,Saint_Lucia|2001|GQ868616|,Suriname|2005|JN379501|,Trinidad_and_Tobago|2003|JN379508|,Barbados|2007|KU509278|,Suriname|2005|KU728209|,Mexico|2006|HM171538|,Saint_Vincent_and_the_Grenadines|2002|JN379490|,USA|2003|FJ390372|,Kenya|2019|MTW|348,Kenya|2019|MTW|362,Kenya|2019|MTW|4157,Kenya|2019|MTW|337,Cambodia|2013|KT758770|,Cambodia|2011|KF543274|,Taiwan|2012|KP176715|,Singapore|2014|KX224286|,Taiwan|2015|MG895235|,Thailand|2016|LC410194|,Taiwan|2016|MG895284|,Laos|2013|KF816163|,Viet_Nam|2013|KP176710|,Thailand|2013|KU509302|,Taiwan|2015|MG895254|,Taiwan|2016|MG895271|,Thailand|2017|LC410195|,Myanmar|2015|KX357894|,Thailand|2010|JF968098|,Thailand|2015|KT758784|,China|2018|MK894340|,China|2019|MN227697|,India|2017|MH594937|,China|2013|KF954946|,Singapore|2012|KX380841|,India|2012|KY978445|,Singapore|2013|KX224279|,Burkina_Faso|2017|MT261972|IP008|E|,Burkina_Faso|2017|MT261975|IP194|E|,Burkina_Faso|2017|MT261976|IP226|E|,Burkina_Faso|2017|MT261977|IP267|E|,Burkina_Faso|2017|MT261973|IP171|E|,Burkina_Faso|2017|MT261974|IP179|E|,Burkina_Faso|2017|MT261978|IP304|E|,Burkina_Faso|2017|MT261979|IP310|E|,Senegal|2009|KU509282|,China|2017|MH010603|,Japan|2010|AB690858|,China|2009|HM466964|,Cote_d'Ivoire|2008|KT187283|,Malaysia|2010|JF968112|,Indonesia|2010|JN575561|,Indonesia|2009|KX646387|,Indonesia|2004|AY858042|,Taiwan|2015|MG895226|,Indonesia|2012|KC589012|,Indonesia|2013|MK629482|,Taiwan|2015|MG895246|,Indonesia|2009|JN568284|,Indonesia|2009|JF968070|,Indonesia|1998|AB189125|,Indonesia|2015|MK629493|,Indonesia|2016|MK629492|,Taiwan|2016|MG895263|,Indonesia|2015|KY499643|,Taiwan|2016|MG895272|,Malaysia|2002|LT898492|,Malaysia|2006|LT898491|,Malaysia|2004|LT898488|,Malaysia|2008|LT898497|,Malaysia|2009|LT898496|,Singapore|2009|JN022605|,Singapore|2009|JN196615|,Australia|2013|KT758787|,Papua_New_Guinea|2016|KY495824|,Australia|2009|JN575579|,Papua_New_Guinea|2010|JN575573|,Papua_New_Guinea|2010|KY794786|,Papua_New_Guinea|2007|KY794787|,Papua_New_Guinea|2008|JN575571|,Fiji|2013|KT758761|,Fiji|2014|KM279399|,Nauru|2014|MG967236|,Nauru|2014|KT758777|,Solomon_Islands|2013|KT758762|,Tonga|2015|KT758782|,Solomon_Islands|2014|KT758778|,Taiwan|2013|MG895194|,Solomon_Islands|2013|KU053466|,Samoa|2015|KT758795|,Cambodia|1999|KF955461|,Cambodia|2005|FJ639727|,Cambodia|2005|KF955333|,Cambodia|2005|GQ868628|,Viet_Nam|2005|JN376773|,Taiwan|1998|KY670634|,Viet_Nam|2001|JN376769|,Viet_Nam|2002|JN376770|,Viet_Nam|2003|JN376771|,Viet_Nam|2004|JN376772|,Cambodia|2001|KF955462|,Viet_Nam|2008|FJ547066|,Thailand|2009|JF968084|,Cuba|2001|KT726355|,Cuba|2002|KT726347|,Ecuador|2000|FJ898457|,Peru|2002|KJ189258|,Peru|2004|KJ189265|,Peru|2005|KJ189299|,Peru|2006|KJ189297|,Peru|2007|KJ189268|,Peru|2008|KJ189287|,Peru|2009|KJ189292|,Paraguay|2007|HQ235027|,Belize|2004|JN379510|,Belize|1998|JN379504|,Jamaica|1998|JN379502|,Aruba|1999|HM348812|,Nicaragua|1998|GQ199886|,Brazil|2003|FJ850079|,Colombia|2001|FJ189450|,Colombia|2001|GQ199891|,Colombia|2002|KX926466|,Colombia|2002|FJ189452|,Colombia|2004|FJ189459|,Colombia|2007|JF804042|,Colombia|2006|FJ204476|,Colombia|2006|HM030551|,Colombia|2005|GQ868577|,Puerto_Rico|2003|MK040396|,Honduras|1995|FJ189469|,Nicaragua|1994|FJ882576|,Puerto_Rico|2000|KF955465|,Puerto_Rico|2001|KF955468|,USA|1998|EU482558|,USA|1999|FJ390375|,USA|2002|EU687196|,USA|2000|FJ547078|,USA|2001|FJ547079|,Saint_Kitts_and_Nevis|2008|KU728211|,Puerto_Rico|2006|KF955456|,USA|2007|EU596493|,USA|2004|FJ373302|,Japan|2008|AB447989|,Cote_d'Ivoire|2008|KT187282|,Togo|2009|KT187281|,Bhutan|2007|FJ606705|,India|2011|KP176704|,India|2013|MH822957|,India|2010|JF968097|,India|2005|JQ922556|,Thailand|2010|JF968092|,India|2009|JQ686071|,India|2010|JQ686075|,Pakistan|2006|KF041259|,Pakistan|2007|KF041255|,Pakistan|2008|KF041254|,Pakistan|2009|KF041258|,Pakistan|2011|KM226344|,Singapore|2007|JN030194|,Yemen|2010|HQ336219|,Somalia|2011|KC848585|,Somalia|2011|KC848589|,Somalia|2011|KC848587|,Somalia|2011|KC848588|,Tanzania|2010|AB549332,Comoros|2010|KT187289|,Madagascar|2010|KT187291.1,Djibouti|2012|KT187295|,Djibouti|2012|KT187294|,Djibouti|2011|KT187292|,Djibouti|2011|KT187293|,India|2016|MG721059|,Australia|2007|JN575578|,Singapore|2007|JN022604|,Singapore|2008|JN030166|,Malaysia|2011|LT898503|,Indonesia|2013|KT758768|,Malaysia|2010|LT898498|,Malaysia|2013|KP176709|,Malaysia|2008|JF968068|,Malaysia|2008|LT898500|,Singapore|2009|JN380810|,Vanuatu|2014|KT758779|,Solomon_Islands|2014|MG967239|,Solomon_Islands|2016|KY495821|,Papua_New_Guinea|2011|KT758738|,Papua_New_Guinea|2014|KT758776|,Australia|2008|JN406515|,Indonesia|2010|KM216738|,Indonesia|2015|KT758794|,Indonesia|2015|LC064747|,Bangladesh|2017|LC436666|,Malaysia|2016|KY495820|,Singapore|2014|KX224276|,Taiwan|2016|MG895276|,Indonesia|2015|KU529754|,China|2016|MF598864|,Viet_Nam|2018|MH594462|,Singapore|2015|KY921906|,Taiwan|2016|MG895278|,Singapore|2013|KX224295|,Taiwan|2014|MG895208|,China|2014|KP191531|,Taiwan|2015|MG895239|,Taiwan|2016|MG895266|,Malaysia|2009|JF968086|,Philippines|2014|KT758781|,China|2010|KU570089|,Philippines|2012|KY851648|,Philippines|2010|JN575570|,Taiwan|2012|MG895185|,Philippines|2011|KT758739|,Singapore|2009|JN030176|,Philippines|2008|JF968055|,Australia|2006|JN575577|,Taiwan|2012|MG895180|,Taiwan|2011|MG895175|,Philippines|2009|JF968083|,Philippines|2013|KT758766|,Philippines|2015|KT825075|,Taiwan|2016|MG895285|,Philippines|2016|KY495822|,Malaysia|2002|LT898507|,Australia|1998|JN575562|,Thailand|1997|JN575575|,Thailand|1994|KJ737429|,Thailand|1997|JN575574|,Thailand|2001|FJ744729|,Taiwan|2013|MG895205|,Taiwan|2013|MG895204|,Taiwan|2011|MG895296|,Taiwan|2014|MG895220|,Taiwan|2011|MG895174|,Viet_Nam|2007|FJ562100|,USA|2005|FJ182011|,Mexico|2003|FJ898440|,Mexico|2006|FJ898441|,Mexico|2007|FJ898442|,Honduras|2013|KY851600|,El_Salvador|2012|JX891664|,Costa_Rica|1995|JF804039|,Nicaragua|2008|GQ199860|,Nicaragua|2008|KF955490|,Nicaragua|2009|JN183884|,Nicaragua|2010|JN000938|,Nicaragua|2009|HQ705609|,Nicaragua|2009|JF920396|,Nicaragua|2009|KF971696|,Nicaragua|2009|HM631861|,Nicaragua|2010|JF920397|,Nicaragua|2010|KF921927|,Mexico|2016|MH936417|,USA|2014|KM458191|,Ecuador|2014|MH346207|,USA|2006|FJ547085|,China|2010|JN029822|,Mozambique|1985|FJ882575|,Sri_Lanka|1989|FJ882571|,Sri_Lanka|1990|FJ189449|,Sri_Lanka|1989|KF955474|,Sri_Lanka|1997|GQ252674|,Sri_Lanka|1993|FJ882573|,Kenya|2019|MTW|4167,Kenya|2019|MTW|359,Kenya|2019|MTW|361,Kenya|2019|MTW|355,Kenya|2019|MTW|34748,Kenya|2019|MTW|344,Kenya|2019|MTW|341,China|2018|MK894339,Kenya|2019|MTW|419,Kenya|2019|MTW|338,Kenya|2019|MTW|34985,Kenya|2019|MTW|339,Saudi_Arabia|2014|KJ830751|,Singapore|2013|KP685235|,Taiwan|2014|MG895222|,Taiwan|2016|MG895267|,Taiwan|2016|MG895287|,Singapore|2011|JN544412|,Sri_Lanka|2006|KU509283|,French_Polynesia|1992|AY744682|,French_Polynesia|1993|JQ650053|,French_Polynesia|1990|AY744680|,French_Polynesia|1990|EU182240|,French_Polynesia|1991|JQ650049|,Cook_Islands|1991|JF804043|,French_Polynesia|1992|JQ650051|,French_Polynesia|1989|EU182242|,New_Caledonia|1989|JQ650061|,French_Polynesia|1996|EU182241|,French_Polynesia|1994|AY744685|,French_Polynesia|1995|JQ650056|,New_Caledonia|1995|JQ650062|,New_Caledonia|1996|JQ650070|,Wallis_and_Futuna|1995|JQ920489|,Samoa|1995|FJ898456|,Indonesia|2008|KC762687|,Malaysia|2012|MF004386|,Indonesia|1998|AB189128|,Malaysia|1991|LT898493|,Indonesia|2010|KT204463|,Indonesia|2013|KY851651|,Indonesia|2016|MH173166|,Indonesia|2010|JF968091|,Malaysia|2014|KT758774|,Indonesia|2008|JN575567|,Singapore|2010|JN030181|,Indonesia|2013|KJ184317|,Taiwan|2013|MG895203|,Taiwan|2013|MG895195|,Australia|2012|KT758743|,Indonesia|2016|KY863456|,China|2010|JN009098|,USA|1963|JQ922554|,Kenya|2019|MTW|3158,Kenya|2019|MTW|364,Philippines|1956|KU050695|,Brazil|2006|JN697379|,China|1980|AF317645,Japan|1973|AB111085,Kenya|2015|MT076953|,Philippines|1964|KM190937|,Kenya|2015|MT076948|,Kenya|2015|MT076951|,Kenya|2015|MT076950|,Bangladesh|2006|JN036388|,Bangladesh|2007|JN036386|,Bangladesh|2008|JN036384|,Singapore|2009|JN030195|,Bangladesh|2009|JF968085|,Philippines|2010|JF968103|,China|2013|KX262919|,China|2013|KM651771|,China|2013|KJ545442|,Laos|2010|KY849775|,Laos|2013|LC147059|,Laos|2013|KF816162|,Myanmar|2009|JF968088|,Taiwan|2013|MG895202|,Thailand|2011|KT758740|,Thailand|2012|MH888332|,Thailand|2013|KT758790|,Malaysia|1987|LT898505|,Malaysia|1988|LT898504|,Malaysia|1990|LT898506|,Malaysia|1995|LT898511|,Thailand|1983|KJ737430|,Thailand|1973|DQ863638|,Cambodia|2008|GU131903|,Cambodia|2007|KF955464|,Viet_Nam|2007|JN376774|,Cambodia|2008|JF968058|,Cambodia|2007|GU131913|,Viet_Nam|2006|EU482458|,Viet_Nam|2007|HQ588141|,Viet_Nam|2010|HQ141582|,Viet_Nam|2012|KY851656|,Viet_Nam|2009|JF968073|,Thailand|2006|JF812103|,Taiwan|2012|MG895183|,Taiwan|2013|MG895197|,Singapore|2013|KX224294|,Taiwan|2012|MG895186|,Thailand|2010|JF968093|,Thailand|2004|JQ993230|

**S3 Table. Dengue isolates used in this study for the complete polyprotein phylogenetic analysis**

Anguilla|2001|FJ898462|,Australia|1998|JN406514|,Australia|2008|JN406515|,Barbados|2007|KU509278|,Bolivia|2011|MH888333|,Brazil|2001|FJ898446|,Brazil|2002|JF808118|,Brazil|2002|JX669490|,Brazil|2003|FJ850079|,Brazil|2003|JF808126|,Brazil|2004|JF808119|,Brazil|2004|JX669498|,Brazil|2006|FJ850089|,Brazil|2006|GU131851|,Brazil|2006|JN697379|,Brazil|2007|GU131869|,Brazil|2007|GU131874|,Brazil|2009|JF808120|,Burkina_Faso|2017|MT261975|,Burkina_Faso|2017|MT261972|,Burkina_Faso|2017|MT261976|,Burkina_Faso|2017|MT261977|,Burkina_Faso|2017|MT261978|,Cambodia|2000|KF955332|,Cambodia|2001|GQ868626|,Cambodia|2002|FJ639722|,Cambodia|2003|FJ639723|,Cambodia|2003|FJ639725|,Cambodia|2005|FJ639727|,Cambodia|2008|GU131946|,China|2009|JF504679|,China|2009|JN662391|,China|2012|KC261634|,China|2013|KF824903|,China|2013|KF954946|,China|2013|MF370226|,China|2015|MN018370|,China|2015|MN018378|,China|2016|MF682970|,China|2016|MN018373|,Paraguay|2007|HQ235027|,Peru|2002|KJ189258|,Peru|2004|KJ189262|,Peru|2005|KJ189293|,Peru|2006|KJ189297|,Peru|2007|KJ189283|,Peru|2008|KJ189287|,Peru|2009|KJ189292|,Philippines|1956|KU050695|,Philippines|1964|KM190937|,Philippines|2008|KU509279|,Puerto_Rico|2000|KF955465|,Puerto_Rico|2001|KF955468|,Puerto_Rico|2006|KF955456|,Saint_Lucia|2001|GQ868616|,Samoa|1995|FJ898456|,Saudi_Arabia|2014|KJ830751|,Senegal|2009|KU509282|,Singapore|2007|GU370053|,Singapore|2012|KX380839|,Singapore|2012|KX380841|,Singapore|2013|KX380842|,Singapore|2015|KY921906|,Singapore|2015|KY921907|,Singapore|2016|MN453624|,Sri_Lanka|1983|GQ199888|,Sri_Lanka|1989|FJ882571|,Sri_Lanka|1993|FJ882573|,Sri_Lanka|1997|GQ252674|,Sri_Lanka|2006|KU509283|,Taiwan|1998|DQ675528|,Taiwan|1999|DQ675533|,Thailand|1983|KJ737430|,Thailand|1994|AY876494|,Thailand|1998|KY586719|,Thailand|2000|KY586794|,Thailand|2001|FJ744737|,Thailand|2002|KY586783|,Thailand|2002|KY586788|,China|2017|MN018389|,China|2018|MK894339|,China|2018|MK894340|,China|2018|MK894341|,China|2019|MN922034|,China|2019|MN964273|,China|2019|MN227697|,China|2019|MN922036|,China|2019|MN922041|,Colombia|2001|GQ199891|,Colombia|2002|GQ868571|,Colombia|2003|FJ898443|,Colombia|2003|GQ868572|,Colombia|2003|GU131951|,Colombia|2004|GU131953|,Colombia|2005|GQ868577|,Colombia|2006|GU131954|,Colombia|2007|GQ868578|,Colombia|2015|MH544650|,Colombia|2016|MH544651|,Cook_Islands|1991|FJ898455|,Cuba|2001|KT726340|,Cuba|2001|KT726356|,Cuba|2002|KT726345|,East_Timor|2005|AB214879|,Ecuador|2000|FJ898457|,French_Polynesia|1989|AY744677|,French_Polynesia|1990|AY744679|,French_Polynesia|1992|AY744683|,French_Polynesia|1994|AY744685|,Gabon|2016|LC379196|,Gabon|2017|LC379197|,Grenada|2002|KF955505|,Guyana|2002|FJ898464|,India|1966|JQ922555|,India|2005|JQ922556|,India|2005|JQ922557|,India|2007|FJ644564|,India|2008|GQ466079|,India|2009|KU509281|,Thailand|2004|KY586730|,Thailand|2004|KY586781|,Thailand|2006|KY586778|,Thailand|2006|KY586817|,Thailand|2008|KU509284|,Thailand|2010|KY586807|,Thailand|2011|KU509280|,Thailand|2012|MH888332|,Thailand|2016|LC410193|,Trinidad_and_Tobago|2002|FJ898459|,USA|1963|JQ922554|,USA|1998|EU482563|,USA|1998|EU482596|,USA|1998|EU529703|,USA|1999|EU529696|,USA|1999|EU687226|,USA|2000|EU529697|,USA|2000|FJ410177|,USA|2001|FJ547081|,USA|2001|FJ547082|,USA|2002|FJ373306|,USA|2002|FJ390373|,USA|2002|FJ478456|,USA|2002|FJ547083|,USA|2003|EU482564|,USA|2003|EU482595|,USA|2003|EU529702|,USA|2003|EU687197|,USA|2003|EU726769|,USA|2003|FJ390371|,USA|2004|EU529705|,USA|2004|FJ024466|,USA|2004|FJ024468|,USA|2004|FJ024470|,USA|2004|FJ182005|,USA|2005|FJ182010|,USA|2005|FJ182038|,USA|2006|EU529692|,USA|2006|EU529699|,India|2013|MH822957|,India|2016|MG721059|,India|2016|MG721061|,India|2017|MH891766|,Indonesia|1998|AB189126|,Indonesia|2004|AY858037|,Indonesia|2004|AY858047|,Indonesia|2007|KC762684|,Indonesia|2007|KC762686|,Indonesia|2008|KC762691|,Indonesia|2016|KY863456|,Indonesia|2016|MH823209|,Kenya|2019|MTW-341, Kenya|2019|MTW-355,Kenya|2019|MTW-359,Kenya|2019|MTW-4167,Kenya|2015|MT076948,Laos|2010/08/05|KY849775|,Malaysia|2012/10/04|MF004386|,Malaysia|2014/12|MH051731|,Malaysia|2014/12|MH051732|,Malaysia|2014/12|MK005258|,Mexico|2006|FJ898441|,Mozambique|1985|FJ882575|,New_Caledonia|1989|JQ920481|,New_Caledonia|1996|JQ920486|,Nicaragua|1998|GQ199886|,Nicaragua|2008|FJ850048|,Nicaragua|2009|HQ705609|,Nicaragua|2010|JF920409|,Nicaragua|2011|KF973481|,Nicaragua|2012|KF973480|,Pakistan|2006|KF041257|,Pakistan|2006|KF041259|,Pakistan|2007|KF041255|,Pakistan|2008|KF041254|,Pakistan|2009|KF041258|,Papua_New_Guinea|2007|KY794787|,Paraguay|2002|JF808123|,Paraguay|2003|JF808129|,USA|2007|EU596492|,Venezuela|2000|FJ639746|,Venezuela|2001|EU529691|,Venezuela|2001|FJ182015|,Venezuela|2002|FJ639775|,Venezuela|2003|FJ639780|,Venezuela|2004|FJ639800|,Venezuela|2005|EU854292|,Venezuela|2005|FJ639803|,Venezuela|2005|FJ639810|,Venezuela|2006|HQ332170|,Venezuela|2007|FJ850109|,Venezuela|2007|FJ898474|,Venezuela|2007|GQ868586|,Venezuela|2010|MH450311|,Viet_Nam|2006|EU482454|,Viet_Nam|2006|EU482457|,Viet_Nam|2007|FJ432728|,Viet_Nam|2008|FJ461322|,Wallis_and_Futuna|1989|JQ920487|,Wallis_and_Futuna|1995|JQ920489|,Thailand|2003|KY586749|,USA|2006|FJ547085|
