# Supplementary material for: March 2019 dengue fever outbreak at the Kenyan south coast involving dengue virus serotype 3, genotypes III and V
Source: PLOS Glob Public Health. 2022 Mar 24;2(3):e0000122. doi: 10.1371/journal.pgph.0000122 (PMC10021577; doi:10.1371/journal.pgph.0000122)
Supplement: Attachment — Submitted filename: Responses to Reviwer(s).docx [file pgph.0000122.s004.docx]

**Responses to the reviewers**

**Editor’s comments**

1. We noticed that you used “data not shown”/"unpublished data" in the manuscript. We do not allow these references, as the PLOS data access policy requires that all data be either published with the manuscript or made available in a publicly accessible database. Please either remove these references, or amend the supplementary material to include the referenced data.

**Thank you for catching these omissions. We have amended the manuscript and provided data in the two instances with “data not shown”. See:**

- **“S1 Figure” on line 277, 282 and 321 in the MS with track changes**
- **“S1 Table” on page 224, and 432 in the MS with track changes**
- **Data availability statement on lines 449 to 451 in the MS with track changes**

**We have no instance of “unpublished data”**

2. We do not publish any copyright or trademark symbols that usually accompany proprietary names, eg (R), (C), or TM (e.g. next to drug or reagent names). Therefore please remove all instances of trademark/copyright symbols throughout the text, including MagPurix Evo® on page 5 and Dengvaxia® on reference 42 and 43.

**-We have now removed all instances of trademark/copyright symbols from the entire Manuscript**

**(Line 126, 128) and in the references 44 and 45 in the MS with track changes**

3. In the online submission form, you indicated that your data will be submitted to a repository upon acceptance. We strongly recommend all authors deposit their data before acceptance, as the process can be lengthy and hold up publication timelines. Please note that, though access restrictions are acceptable now, your entire data will need to be made freely accessible if your manuscript is accepted for publication. This policy applies to all data except where public deposition would breach compliance with the protocol approved by your research ethics board. If you are unable to adhere to our open data policy, please kindly revise your statement to explain your reasoning and we will seek the editor's input on an exemption. Please be assured that, once you have provided your new statement, the assessment of your exemption will not hold up the peer review process.

**-All sequence data generated from this work is now publicly available in the GenBank nucleotide sequence repository as indicated in the data availability statement (Line 449 to 451) in the MS with track changes**

Additional Editor Comments (if provided):

The manuscript by Muthanje et al. reports the use of genomic data to evaluate a Dengue Fever (DF) outbreak in Kenya’s south coast in March 2019. Recurrent epidemics have been reported from the coastal areas of Kenya (Agha et al. 2019), with the latest warning having issues as recently as May of 2021 (https://reliefweb.int/report/kenya/kenya-dengue-fever-outbreak-emergency-plan-action-epoa-n-mdrke048). Genomic data from previous outbreaks (2011 and 2017) has been previously analysed and reported of Dengue Virus (DENV) serotype 2 (DENV-II) in the same region (Langat et al. 2020), with a particular interest in the co-circulation of two different Cosmopolitan genotype lineages (DENV-II genotype Cosmopolitan, also named DENV-II genotype II). Unlike the previously reported studies, this study reports the causative agent of the outbreak as DENV serotype 3 (DENV-III), belonging to the genotypes III and V. Unlike serotype classification, which differs from each other by 25–40 % at the amino acid level and can be easily detected through the qPCR targeting the targets the Capsid (C) and pre-Membrane (preM) region, the genotyping is obtained though full genome homology, vary by up to 3 % at the amino acid level (Diamond and Pierson 2015). High-throughput sequencing (HTS) technologies allow the simultaneous identification and characterization by serotyping and genotyping of DENV cases at the nucleotide level in a single methodological step, with previous studies having demonstrated that complete viral sequences can be obtained directly from patient sera using a shotgun metagenomics approach (Yozwiak et al. 2012). Therefore, the methodology used in this study has been successfully used in similar studies, with success in inferring the genetic relationship between DENV strains.

Major Comments

1. The authors started with a collection of 37 plasma samples from febrile patients, of which 21 were confirmed through qPCR positive for DENV-3 (56.75%). This is a low number of DENV samples, and no context is provided of the timeframe and geographical distribution of these samples. The only information provided was they were collected in March of 2019 from the Kenyan south coast, but no indication if they were from the same hospital or medical care centre was provided.

**The samples used in our study were collected under a protocol as part of an ongoing acute febrile illness surveillance. Fever is the only eligibility criterion. Enrolled subjects are tested for diverse endemic pathogens by targeted PCR. Those that are negative are screened by WGS. The DENV PCR positivity rate was 56.75%. It goes to show that, even during an outbreak, not all patients reporting to the hospital with fever will have DF. Therefore, the DENV negative samples could have had other etiologies, or that the viremia was too low to be detected by the qPCR assay used.**

**We have now provided more details about the outbreak time frame, region and hospital of sample collection. See “Study Design section” (starting from line 116 of the MS with track changes). Its states:**

**“37 plasma samples were collected during March 2019 DF outbreak from febrile patients seeking hospital care at Mtongwe Naval Base Hospital, Mombasa County. The samples were collected under an ongoing acute febrile illness surveillance protocol that was reviewed and approved by the Kenya Medical Research Institute’s Scientific and Ethical Research Unit (KEMRI SERU #1282) and the Walter Reed Army Research Institute’s Human Subject Protection Branch (WRAIR #1402). A written informed consent was obtained from each participant”.**

2. Of the 21 DENV-3 positive samples, the complete genomes were obtained only in 4 samples (19.04%). The authors describe the workflow used in the CLC Genomics Workbench, following the standard procedure of read filtering based on quality and de novo assembly. No information on the thresholds used for quality filtering is indicated, nor the average quality score of the remaining sequences. The authors also describe the use of reference guided assembly, without indicating its purpose. It is therefore unclear if the assemblies are obtained through de novo assembly or through guided assembly, and the caveats of consensus-based assembly are also not indicated. In previous studies, full DENV genomes were assembled even in samples containing as low as 960 DENV reads (Mendes et al. 2020).

**We agree with the reviewer that our methodology was too abridged. We have provided more details on assembly methods used to obtain genomes from shotgun (whole genome) and targeted (E gene envelop) sequencing. For the shotgun approach, the workflow first involved *de novo* assembly of sequence reads. This aided identification of DENV genotype that was most homologous to genomes in our study. Reference guided assembly was thereafter used to process the shotgun and targeted sequence reads in CLC Genomics. This section has now been expanded and additional information on thresholds used for quality filtering and consensus calling provided (see section titled “Sequence assembly and phylogenetics”, lines 145 to 160 in the MS with tracked changes).**

**We acknowledge that consensus sequences generated from reference guided assemblies could suffer a bias and inaccuracies due to the choice of the reference. However, these inaccuracies are negligible when the divergence between the reference and study sample is small, or the reference and study sample are within the same species (Bohling 2020). For example, the genotypes of DENV-3 do not exceed a nucleotide divergence of 6% (Chen and Vasilakis 2011, Sim et al 2016). Furthermore, a consensus built on protein coding genes tend to be accurate given their conserved nature, unlike more rapidly diverging genomic features such as transposable elements, or other non-coding region (Daren et al 2014). This study built the consensus on the complete polyprotein, and on the E gene protein of DNV3.**

**Thanks for pointing us to the Mendes et al., 2020. From the paper, it was difficult to draw any information on sample 91-0106 that contained 960 DENV reads but successfully assembled a complete genome. Information on sample matrix used and whether it was from PCR amplicons or plaque counts from viral culture would be helpful. Furthermore, the sample is missing from the supplementary information TableS1 that details the clinical samples sequenced using shotgun metagenomics. This notwithstanding, the number of reads presents in a sample does not explicitly mean sufficient read breadth and depth during genome assembly. Full genome recovery could be affected by a wide range of factors including high host vs virus genome in the sample, disproportionate sequencing across the genome.**

**Nevertheless, we have added a sentence in the discussion section that speculates on why not all samples were sequnceable. It states:**

**“We speculate that the failure to obtain WGS when Ct values were within “sequenceable” range was due to factors such as sample integrity and/or presence of background genomes from the host, or commensal microbiome, as has been alluded to in a previous [58]”. (Lines 362-367 in the MS with track changes).**

3. The CT values of the 21 DENV-3 positive samples ranged between 20 and 39. According to previous studies with the typing kit used, the pan-alphavirus assay had a sensitivity range of 10–25 copies per reaction depending on the viral strain (Garae et al. 2020). The authors relate the high CT values with the unsuccess of the assembly of the viral genomes. It is important to include controls to clearly associate viral load with viral sequences obtained, and the success of the assembly.

**It is not clear why the reviewer would want us to include controls with known viral load (copy numbers). The intent of the study was not to quantify the assay sensitivity or quantify viral load in study samples. We highlighted Ct values because we noticed that the success of sequencing was correlated to Ct values. The inverse relationship between Ct values and viral load is very well established. Anyhow, we have added a statement in the discussion that says :** **To better quantify the inverse relationship of Ct values and recovery of complete genomes, future studies would benefit from the use of control/s with known copy numbers” (Lines 361-367 in the MS with track changes).**

4. The Kenyan DENV-3 dataset comprising the full DENV genomes assembled (n=4) was supplemented with 221 sequences from VIPR, filtered by geographical location, year of collection, genotype and presence of the full envelope protein. It is unclear why the query was not limited to full sequences, as genotyping is performed on the homology of the full coding sequence. Additionally, no indication of the values for the filters was made available, which strongly hinders the replication of the search query. A full list of accession numbers should also be made available as supplemental material.

**-The reviewer’s question on why sequence querying was not limited to full sequences is probably referring to the Envelop gene and not the full DENV genomes (n=4). For the 4 full genomes, the query was based on full sequences. For the E envelop gene, the query was based on full envelop gene. To make it clearer, we have expounded and clarified the subsampling process for the whole genome and envelop gene phylogenies. We have also expounded on the subsampling algorithm used. We also agree with the reviewer on the need to provide a full list of accession numbers (now provided as Supplementary table 2. See lines 186 – 194 in the MS with track changes)**

5. It is stated by the authors that the multiple sequence alignments of the complete DENV coding sequences and envelope protein region were manually edited using CLC Genomics Workbench, no information is stated on the purpose or type of edition that was performed.

**-Thanks for catching this omission. The statement now reads “Alignments were manually edited using CLCGx v8.5.1 in order to fix any misalignments that might have been inadvertently introduced by the alignment algorithm”. (see Line 197-198 in the MS with track changes)**

6. The conclusions drawn from the co-circulation of genotype III and V are drawn from the send of 17 envelope sequences obtained from the original 21 Kenyan sample dataset. This includes the 4 assembled complete genomes. The global dataset obtained from VIPR includes 383 sequences, which is assumed that also are partial sequences. Given that only envelopes sequences are used, the genotype classification is greatly hindered as other genetic regions, such as NS1, NS3, and NS5 have been shown to exhibit higher phylogenetic support (Cuypers et al. 2018). Therefore, whole-genome sequences provide superior classification precision and are particularly relevant when assessing the emergence and co-occurrence of DENV strains belonging to the same serotype, but different genotype. This is also relevant in the molecular clock analysis performed, where the 398 envelope sequences were used to estimate the evolutionary rate of the dataset. Limiting this analysis to a region and not the full genomic sequence of the DENV greatly impacts the results obtained.

**As mentioned by Cuypers et al, 2018 (cited by reviewer),** **the DENV genotype nomenclature and classification system lack consistency. And it appears as though other genetic regions, such as NS1, NS3, and NS5 exhibit higher phylogenetic support (Cuypers et al. 2018). But, as mentioned by Cuypers, envelope gene is the most often used for classification purposes, and for a good reason: its historical role in diagnosis, influence on DENV infectivity and immunogenicity****, including the fact that it’s a DENV vaccine candidate.** **Importantly, E gene sequences are the most available in public repositories. We have included a statement to acknowledge the utility of other genes in the phylogeny of DENV, and also made a case for the use of E gene. (Lines 377-395 in the MS with track changes)**

7. Whole-genome targeted sequencing of DENV samples has been successfully used to obtain complete sequences from clinical samples (Parameswaran et al. 2017) and is a viable and cost-effective option for obtaining reliable data for phylogenetic inference and should be preferred to methods selecting a particular region of the genome.

**We have now addressed this concern (Line 377-395 in the MS with track changes)**

8. DENGVAXIA vaccine contains chimeric yellow fever dengue viruses with serotype 1 to 4. It is constructed using recombinant DNA technology by replacing the sequences encoding the pre-membrane (prM) and envelope (E) proteins in yellow fever(YF) 17D204 vaccine virus genome with those encoding for the homologous sequences of dengue virus serotypes 1, 2, 3, and 4, respectively. The analysis performed only contained the DENV genomic region coding for the envelope. The pre-membrane region should not be excluded when analysing the potential loss of efficacy of the vaccine in the Kenyan DENV-3 samples.

**We agree with the reviewer about this limitation. Unfortunately, our E gene sequences did not include the pre-membrane region. A recent study by Dias M, Pattabiraman C, Siddappa S et al, 2019 used a similar comparative approach to ours (i.e. minus PrM). We have added a sentence in the discussion to indicate this potential shortcoming of not including prM (Line 433-436 in the MS with track changes).**

9. Supplemental material should be made available for peer review as it might contain or might be missing important information for the comprehension of the manuscript.

**-This has now been included. (See lines 224, 277, 282, 321, 432 in the MS with track changes)**

Minor Comments

1. In accordance with the previous comments, the title should be changed to “Dengue fever outbreak at the Kenyan south coast involving serotype 3”.

**We deliberately chose to include genotypes III and V in the title since these genotypes constitute the new findings in our study. We have however revised the title to read:**

**“March 2019 dengue fever outbreak at the Kenyan south coast involving dengue virus serotype 3, genotypes III and V”**

2. Support should be added to the phylogenetic trees (Figure 1, Figure 2, and Figure 3)

**The reviewer could have missed details in the figure legends. The figures are color coded: Blue = Genotype III, Magenta = Genotype IV, Grey = Genotype I, Black = Genotype V and Green = Genotype II. These details are provided in all the figure legends. We have however included details that may have been missing in the figure legends: Fig 1 (lines 262-266 , Fig 2 (lines 283-290) and Fig 3 (lines 307-314 in the MS with track changes).**

3. In the Introduction, a few DENV serotypes and genotypes are stated by name but no clear description of the DENV classification, both at the serotype and genotype level, is presented.

**-We thank the reviewer for catching this omission. We have now included the genotypes of the other dengue serotypes. [Lines 56 – 65 in the MS with track changes].**

4. An appropriate reference should be added to support the statement in the introduction that “The serotype share 65-70% amino acid similarity”.

**-This citation has now been added in the text [Line 60 in the MS with track changes].**

5. In the introduction, there’s a missing space between “DENV-1-3” and the reference [19, 20].

**-We have corrected this omission [Line 77 in the MS with track changes]**

6. In the introduction, the following phrases are overly complicated and very confusing to read: “This could be due to an increase in [the] use of DF diagnosis, and/or DF surveillance. It could also be due to an increase in DENV in the population due to increased vector-human interaction and/or importation driven by an increase in transhuman movements from endemic countries”.

**-Thanks for pointing this out. The confusion has now been resolved by restructuring the statements. The statement now reads:**

**“In the past decade, there has been increased reports of DF outbreaks at the Kenyan coast probably due to improved diagnosis, surveillance and increased spread of virus as a result increased trans human movement from endemic to non-endemic areas”. [Line 98-103 in the MS with track changes]**

7. In the methods section, Homology estimates of the DENV-3 env protein to the Thailand DENV-3 strain used in the approved Dengvaxia vaccine, it is stated that “comparisons were also made to other African DENV-3 sequences available in GenBank”. The accessions of the sequences used should be stated.

-**This is now shown in “Supplementary table 1 [Lines 222-224 in the MS with track changes]**

8. A small typo is present in the second phase of the “DENV-3 from the 2019 Coastal Kenya outbreak has been in circulation since 2015” section, where ‘.,’ are present.

**-This has been rectified [Line 293 in the MS with track changes]**

[Note: HTML markup is below. Please do not edit.]

Reviewers' comments:

Reviewer's Responses to Questions

Comments to the Author

1. Does this manuscript meet PLOS Global Public Health’s publication criteria? Is the manuscript technically sound, and do the data support the conclusions? The manuscript must describe methodologically and ethically rigorous research with conclusions that are appropriately drawn based on the data presented.

Reviewer #1: Yes

Reviewer #2: Yes

2. Has the statistical analysis been performed appropriately and rigorously?

Reviewer #1: N/A

Reviewer #2: Yes

3. Have the authors made all data underlying the findings in their manuscript fully available (please refer to the Data Availability Statement at the start of the manuscript PDF file)?

The PLOS Data policy requires authors to make all data underlying the findings described in their manuscript fully available without restriction, with rare exception. The data should be provided as part of the manuscript or its supporting information, or deposited to a public repository. For example, in addition to summary statistics, the data points behind means, medians and variance measures should be available. If there are restrictions on publicly sharing data—e.g. participant privacy or use of data from a third party—those must be specified.

Reviewer #1: Yes

Reviewer #2: Yes

4. Is the manuscript presented in an intelligible fashion and written in standard English?

PLOS Global Public Health does not copyedit accepted manuscripts, so the language in submitted articles must be clear, correct, and unambiguous. Any typographical or grammatical errors should be corrected at revision, so please note any specific errors here.

Reviewer #1: Yes

Reviewer #2: No

5. Review Comments to the Author

Please use the space provided to explain your answers to the questions above. You may also include additional comments for the author, including concerns about dual publication, research ethics, or publication ethics. (Please upload your review as an attachment if it exceeds 20,000 characters)

**Responses to Reviewer 1**

Reviewer #1: The subject of the manuscript, the observation of dengue virus serotype 3 causing the outbreak at the Kenyan south coast is an important notification, as like authors also state in the manuscript, the recent outbreaks in Kenya have typically been caused by DENV-2. The article is well written and pleasant to read. This article provides important information about DENV-3 strains circulating in Kenya. There are however, issues that should be addressed in my opinion.

Specific comments

Title

I would indicate the year of the outbreak already in the title

**-We agree with the reviewer. Month and year have now been added to the title**

Abstract

The number of patients would be nice to be stated already in the abstract

**-This has now been added [Line 19 in the MS with track changes]**

Line 19 please change RNA was isolated to RNA was extracted

**-Thank you. The change has been made (line 20 in the MS with track changes)**

Introduction

Line 42 Flavivirus is genus, Flaviviridae family

**-This has been changed [Line 45 in the MS with track changes]**

Line 42 please correct Ae. Albopictus

**-This has been corrected [Line 45 in the MS with track changes]**

Line 43 Ae. aegypti and Ae. albopictus are not globally distributed. The distribution area of especially Ae. aegypti is limited to tropical and subtropical regions. Please correct.

**-This has been corrected (line 46 in the MS with track changes)**

Line 46 Authors state that DENV E gene is more conserved. This is now a desultory remark as the authors do not explain more conserved compared to what?

**-Thank you. The word conserved has been deleted (lines 49-50 in the MS with track changes)**

Line 57, remove for instance, that is not needed.

**-The statement has been revised but we would like to keep the the words “for instance” (lines 61-62 in the MS with track changes).**

Line 72-73 The sentence about pathophysiology of dengue virus infection needs to be rephrased. Even though ADE is strongly associated with severe cases of DENV infections, there are cases of severe dengue also with primary infections.

**-We have now rephrased this statement to include the word “mostly associated” (lines 68-71 in the MS with track changes)**

Line 80 Please add reference to Aedes aegypti abundance at the coast and in Busia

**- Thank you. We have referenced this statement (see reference 31, lines 93 in the MS with track changes)**

In general: Please describe the outbreak, when where, how many cases, reference

**- This request was also made by 2^nd^ reviewer. We have now provided more details about the outbreak time frame, region and hospital of sample collection. See “Study Design section” (starting from line 115 to 122, in the MS with track changes). Its states:**

**“37 plasma samples were collected during the March 2019 DF outbreak from febrile patients seeking hospital care at Mtongwe Naval Base Hospital, Mombasa County in March 2019 from febrile patients with suspected cases of DF. The samples were collected under an ongoing acute febrile illness surveillance protocol that was reviewed and approved by Kenya Medical Research Institute’s Scientific and Ethical Research Unit (KEMRI SERU #1282) and the Walter Reed Army Research Institute’s Human Subject Protection Branch (WRAIR #1402). A written informed consent was obtained from each participant”.**

Materials and methods

Line 100 please rephrase “The study used 37 plasma samples”.

**– This has been rephrased (lines 115 to 122 in the MS with track changes)**

In Study design please tell abit more about the patient material.

**– We have now provided more details about the outbreak time frame, region and hospital of sample collection. See “Study Design section” (starting from line 115 in the MS with track changes). Its states:**

**“37 plasma samples were collected during the March 2019 DF outbreak from febrile patients seeking hospital care at Mtongwe Naval Base Hospital, Mombasa County in March 2019 from febrile patients with suspected cases of DF. The samples were collected under an ongoing acute febrile illness surveillance protocol that was reviewed and approved by Kenya Medical Research Institute’s Scientific and Ethical Research Unit (KEMRI SERU #1282) and the Walter Reed Army Research Institute’s Human Subject Protection Branch (WRAIR #1402). A written informed consent was obtained from each participant”.**

Were the patients diagnosed with dengue virus infection?

**– At the hospital, the patients were suspected to have DF based on the physician assessment and increased number of cases with similar signs**. **The clinician diagnosis indicated in the case report form was “suspected dengue fever”.**

The sample size is quite small so were there other inclusion criteria than just suspected dengue virus infection?

**– The samples were collected under an ongoing acute febrile illness surveillance protocol. Inclusion criteria is fever.**

What kind of disease/symptoms did they have?

**– Unfortunately, the clinicians did not provide detailed disease/symptoms description. The information captured in the case report form was “suspected dengue fever”.**

The hospital the samples were from is written in the manuscript but it would be good also to describe the cities/municipalities the patients were from.

**–This information has now been provided. See lines 115 to 122 in the MS with track changes.**

Line 121 it is more common to talk about E gene than env gene.

**-We have now replaced all instances of env gene with E gene**

In general few points about Materials and methods. The authors should describe how they have confirmed the validity of the results. For example data of positive controls in PCR and how they have excluded the possibility of contamination.

**-Thanks for catching this omission. We have now included a statement on how potential PCR contaminations were tracked [Line 150 in the MS with track changes].**

Discussion

Line 269 please change DF infection to dengue virus infection

**-We have rephrased the sentence to say ……**”**the mosquito vectors responsible for transmitting DENV within countries neighboring the Indian Ocean” (Lines 333-334 in the MS with track changes).**

Lines 295-297 “Judging from this data, it is clear that, apart from viral load, other factors such as sample integrity and/or presence of other contaminating genomes are crucial for the success WGS.” The authors should explain what they mean with contaminating genomes regarding to their sample materials/analyses.

**-We have rephrased the statement to say:**

**“We speculate that the failure to obtain WGS when Ct values were within sequenceable range was due to factors such as sample integrity and/or presence of background genomes from the host or commensal microbiome, as has been alluded to in a previous reports [See lines 361 – 366 in the MS with track changes]**

I would like the discussion to include more analyses of the relation of the sequences produced in this study and previous DENV-3 sequences from Kenya.

**-This is a good suggestion. Unfortunately, there are very few Kenyan DENV-3 sequences in genome repositories. For instance, there are only one complete genome from Kenya (shown in Figure 1) and four E gene sequences shown in Fig 2). The one full sequence and the four E gene sequences belong to genotype V. On page 17 (lines 406-417 in the MS with track changes), we have discussed the relationships of our DENG-3 sequences and those in previous studies. We say the following:**

**“The tree topology of the genotype V clade (Figure 3, panel C) has two lineages with their TMRCA dating back to 1949 (95%HPD: 1945-1953). Lineage one contained two Kenyan south coast samples from this study (MTW-3158 and MTW-364) while lineage two contained four previously reported genotype V that were collected in 2015 from Western Kenya [25] (MT076948, MT076951, MT076950 and MT076953). It is tempting to speculate that the two divergent genotype V lineages are in circulation in Kenya, probably geographically split into a coastal and Western Kenya population. We are cognizant of the limitation of this speculation, especially due to under sampling of DENV-3 genotype V in the country as well as globally. Currently, apart from the recent Brazil (2006) and Kenyan samples (2015 and 2019), the clade is populated by old viruses, the most recent being from China (1980). Members of the genotype V lineage are rarely reported and have previously even been considered extinct”.**

**Really, there is no too much else to say.**

Figures and tables

Figure 2 Please mark genotypes to the figure. The large figure’s resolution is not good.

**- The reviewer could have missed details in the figure legends. As indicated in the figure legends, the figures are color coded: Blue = Genotype III, Magenta = Genotype IV, Grey = Genotype I, Black = Genotype V and Green = Genotype II. These details are provided in all the figure legends. We have however included details that may have been missing in the figure legends: Fig 1 (lines 262-266 , Fig 2 (lines 283-290) and Fig 3 (lines 307-314 in the MS with track changes).**

**Figure legibility can be increased by zooming out. We will make sure that the resolution meets the journal requirement.**

General comments

Based on the results it might be quite strong statement to say that the virus is of Pakistani origin. It might be good to discuss the Asian origin in more general.

**-We have toned down the statement. We now say that the Kenyan DENV-3 share** **genealogy with those from Pakistan. (See lines 402 and lines 439 in MS with tracked changes).**

Comparison of the Kenyan sequences to the Dengvaxia is a bit irrelevant in my opinion. It is not stated which genotype the DENV-3 used in the vaccine is and it is obious that there are differences if the genotype is not the same. And as it is stated in the manuscript, the differences are the same as with previous virus strains from Kenya. To my knowledge, the vaccine is not in use in Kenya. So, I would leave this comparison and discussion out of the article.

**We humbly beg to differ with the reviewer on this. The whole point of comparing Kenya DENV-3 envelope proteins to those used in Dengvaxia vaccine is to identify matches/mismatches that could help predict why the vaccine could fail to protect against strains circulating in Kenya. We know that the vaccine has limited efficacy, and part of the poor efficacy could be due to differences in the antigenic sites. It behooves the vaccine manufactures to use antigenic sites that are broadly represented in all genotypes so that the vaccine can stand a better chance of being broadly protective. We identified 23 amino acid variances between DENV-3 E sequences in the study samples and the DENV-3 strain from Thailand that is used in the Dengvaxia tetravalent vaccine. A recent study (Dias et al., 2019) found similar variances in the envelope protein of Indian DENV-3 strains compared to Thailand strain used in the Dengvaxia vaccine.**

**Responses to reviewer 2**

Reviewer #2: Line 1: The Kenyan south coast is not defined. Where does it start and where does it end? If it is not properly defined, I recommend to the authors to mention specific locations where the study was undertaken in the title. I would also propose to refer to the serotypes and genotypes as being those of dengue virus (DENV).

**- It will be hard to incorporate a definition of the Kenyan southern coast in the title. In the method section, we have provided more information on the study site. (See lines 115-122 in MS with track changes).**

Line 4: Affiliation #1 is missing the city, while this is indicated in affiliation #2.

**-City information has now been added**

Line 9: Why is superscript #2 is indicated along with the corresponding author’s name?

**-This has been removed**

Line 14: Reconsider your reference to slave trade. Is it worthy mentioning and reminding the reader of this?

**-The reference to 15^th^ Century slave trade era is an important mention because it gives a historical context and gives a clue to how DENV could have spread in slave trade ships across the world.**

Line 18: I recommend including sample size. All of a sudden the numbers start in the results in line 23. Refer to line 177.

**-The sample size has now been added (see lines 19 in MS with tracked changes)**

Line 20: Define all abbreviations at first mention throughout the text. qPCR has. not been defined at first mention.

**-Thank you for catching this omission. We have defined all abbreviations at first mention throughout the MS.**

Line 21: “whole genomes” of what?

**-This has been collected to DENV whole genomes (see lines 23 in the MS with track changes)**

Line 33:”corruption” is a very strong word. Consider an alternate word.

**-It’s intended as a pun, and really, it’s a corruption of the original words. We prefer to keep it.**

Line 35: Was it Tanzania or Tanganyika at that time?

**- We have added the word Tanganyika (see line 38 in the MS with track changes)**

Line 42: Does dengue virus belong to “Flavivirus”? Kindly indicate an appropriate family.

- **Thank you again for catching this mistake. This has now been corrected to Flaviviridae (see line 45 in MS with tracked changes).**

Line 50: What is dengue infection? Do you mean dengue virus infection?

**-This has been corrected to read dengue virus infection (see lines 54 in the MS with track changes)**

Line 53: Please read and cite et al., Viruses. 2021 Mar 24;13(4):536. doi: 10.3390/v13040536.

**-Thank you for directing us to this review. We were aware of it, but had missed to cite it. We have read the publication and included it in the reference list (see lines 57, reference “11” in the MS with track changes)**

Line 63, line 68: Add space between the ending of a sentence and the references.

**-This has now been addressed. See lines 76 and 81 in the MS with track changes**

Line 72: You have previously used DF for dengue. Ensure consistency.

**Thanks again. The use of DF for Dengue Fever and DENV for Dengue Virus is maintained in the whole MS**

Line 88: What about the role of imported tyres.

**-This sentence has been re-written to read:**

**“In the past decade, there has been increased reports of DF outbreaks at the Kenyan coast probably due to improved diagnosis, surveillance, and enhanced spread of the virus as a result of the ever increasing trans human movement from endemic to non-endemic areas”. (see lines 97-102, in MS with tracked changes)**

Line 100: What is the demography of study participants? How were the subjects selected? What was the case definition used? Was there status for other febrile illnesses checked?

**This question was also asked by another reviewer. The samples used in our study were collected under a protocol as part of an ongoing acute febrile illness surveillance. Fever is the only eligibility criterion. Enrolled subjects are tested for diverse endemic pathogens by targeted PCR. Those that are negative are screened by WGS. The DENV PCR positivity rate was 56.75%. It goes to show that, even during an outbreak, not all patients reporting to the hospital with fever will have DF. Therefore, the DENV negative samples could have had other etiologies, or that the viremia was too low to be detected by the qPCR assay used.**

**- We have now provided more details about the outbreak time frame, region and hospital of sample collection. See “Study Design section” (starting from line 115 of the MS with tracked changes). Its states:**

**“37 plasma samples were collected during the March 2019 DF outbreak from febrile patients seeking hospital care at Mtongwe Naval Base Hospital, Mombasa County. The samples were collected under an ongoing acute febrile illness surveillance protocol that was reviewed and approved by Kenya Medical Research Institute’s Scientific and Ethical Research Unit (KEMRI SERU #1282) and the Walter Reed Army Research Institute’s Human Subject Protection Branch (WRAIR #1402). A written informed consent was obtained from each participant.”.**

Line 108: Include location (city) for the manufacturer. Do this throughout the text.

**-This has now been rectified. See line 125 in the MS with tracked changes)**

Line 114: Please check whether abbreviations at the beginning of the sentence is allowed.

**-The word cDNA is acceptable as is.**

Line 119: italicize gene names.

**-Following suggestion by another reviewer, we have now replaced the *env* gene with the more commonly used abbreviation “E gene” to represent the Dengue Envelop gene.**

Line 124: Describe how nested PCR approach was used?

**-The primary and secondary PCR used the same thermal cycling regimen and reaction components. Since the primary PCR approach is already provided, there is no need for duplicity. We therefore used the statement “Thermal cycling conditions for the first and second PCR were similar”. The statement says:**

**“Thermal cycling conditions for the first and second PCR were similar and included initial denaturation at 95°C for 1 min, 35 cycles of denaturation at 95°C for 1 min, annealing at 56°C for 30 sec, extension at 72°C for 1 min and final extension at 72°C for 5 min. (see lines 147-149 in MS with tracked changes)**

Line 140:is it blastn not supposed to be italicized?

**-Thanks for catching the error. We have now changed blastn to “BLASTn” following a random review of multiple papers citing blastn. (see line 166 in the MS with track changes)**

Line 145: Define abbreviation VIPR

**-Done (see line We have now defined VIPR at the first mention (lines 102-103) and thereafter used abbreviation (lines 185, 188, and 387) in MS with tracked changes)**

Line 176: Refrain from concluding statements in the titles. Correct this throughout.

**With due respect, we prefer a statement that sets the stage for the data being presented**

Line 178: why use short cuts like 4/21?

**-This is an acceptable style of writing fractions**

Line 183 – 188: Discuss the ct value inconsistency in the Discussion. Follow guidelines for formatting Tables.

**This is now addressed [see lines 360-366 in the MS with track changes]**

Line 245: There is a repeated word.

**-Thank you. This has been corrected (see line 306 in MS with tracked changes)**

Line 295: Consider adding references to your discussion.

**-This has now been addressed [see new reference 58, lines 361-366 in the MS with track changes]**

Line 337: Can this be proven from the outbreak samples during 2015?

**-The number of publicly available DENV-3 genomes from East Africa is limited, and if the situation was different, more DENV-3 genomes would help increase the resolution of the time trees and clarify the phylodynamics of DENV-3. In the paragraph, we have included a disclaimer citing under sampling of genomes as a study limitation (see lines 418-425 in the MS with track changes MS).**

6. PLOS authors have the option to publish the peer review history of their article (what does this mean?). If published, this will include your full peer review and any attached files.

Do you want your identity to be public for this peer review? If you choose “no”, your identity will remain anonymous but your review may still be made public.

For information about this choice, including consent withdrawal, please see our Privacy Policy.

Reviewer #1: No

Reviewer #2: Yes: Gerald Misinzo

[NOTE: If reviewer comments were submitted as an attachment file, they will be attached to this email and accessible via the submission site. Please log into your account, locate the manuscript record, and check for the action link "View Attachments". If this link does not appear, there are no attachment files.]

While revising your submission, please upload your figure files to the Preflight Analysis and Conversion Engine (PACE) digital diagnostic tool, https://pacev2.apexcovantage.com/. PACE helps ensure that figures meet PLOS requirements. To use PACE, you must first register as a user. Registration is free. Then, login and navigate to the UPLOAD tab, where you will find detailed instructions on how to use the tool. If you encounter any issues or have any questions when using PACE, please email PLOS at figures@plos.org. Please note that Supporting Information files do not need this step.

In compliance with data protection regulations, you may request that we remove your personal registration details at any time. (Remove my information/details). Please contact the publication office if you have any questions.
